# Patients with Autoimmune Thyroid Diseases Have Higher Prevalence of Positive Antiphospholipid Antibodies: A Systematic Review and Meta-Analysis

**DOI:** 10.1155/2022/8271951

**Published:** 2022-08-27

**Authors:** Difei Lu, Zhibo Song, Ying Gao, Junqing Zhang, Xiaohui Guo

**Affiliations:** ^1^Department of Endocrinology, Peking University First Hospital, Beijing, China; ^2^Department of Rheumatology and Clinical Immunology, Peking University First Hospital, Beijing, China

## Abstract

**Introduction:**

Autoimmune thyroid diseases (AITDs) and antiphospholipid syndrome (APS) are commonly seen in childbearing-age women and can lead to recurrent miscarriages. Studies with a relatively small sample size were conducted and concluded inconsistent results on the relationship between AITDs and APS or the presence of antiphospholipid (aPL) antibodies. This meta-analysis aimed to provide evidence on the possible association between AITDs and APL using primary data from all relevant studies.

**Methods:**

Literature databases including PubMed, Embase, and Cochrane were searched from the time when the databases were established to March 2022. A total of 7 studies that met all inclusion criteria were selected in this meta-analysis, with 475 AITD patients and 764 healthy controls. After data extraction, the pooled odds ratio (OR) and the 95% confidence interval (95% CI) were calculated.

**Results:**

The risk of positive APL in AITD patients was approximately 3 folds higher in AITD patients compared with those in healthy controls (OR 3.41, 95% CI 2.29–5.08, *p* < 0.001). There was no significant association between the risk of Graves' disease (GD) and positive APL (OR 9.3, 95% CI 0.10–824.73, *p*=0.33). The risk of positive APL in patients with Hashimoto's thyroiditis (HT) increased over 2 folds compared with healthy controls (OR 3.35, 95% CI 1.55–7.23, *p*=0.002).

**Conclusions:**

The result of this meta-analysis proved that the presence of APL was significantly increased in patients with AITD compared with healthy individuals, especially in patients with Hashimoto's thyroiditis.

## 1. Introduction

Autoimmune thyroid disorders (AITDs), including Graves' disease (GD) and Hashimoto's thyroiditis (HT), are one of the most common organ-specific autoimmune diseases. HT manifests as clinical hypothyroidism due to thyroid autoimmunity (TAI), in other words, the autoantibodies against thyroglobulin and/or thyroid peroxidase. It was reported that HT was a T helper (Th) cell-mediated autoimmune disease, which resulted in diffuse lymphocytic infiltration of the thyroid gland [1]. The main feature of Graves' disease is the production of autoantibodies against the thyroid-stimulating hormone receptor, which induces excessive secretion of thyroid hormone. Both GD and HT lead to a higher risk of infertility and poor outcomes during pregnancy, and women with AITD are prone to receiving assisted reproductive technology (ART) [2]. However, possible mechanisms of reproductive failure due to AITD still remain unclear.

Antiphospholipid syndrome (APS) is an autoimmune disease characterized by recurrent thrombosis and miscarriage due to the presence of antiphospholipid (aPL) antibodies. APS consists of primary conditions or secondary to other underlying autoimmune diseases, most commonly systemic lupus erythematosus (SLE). A series of studies have proved the existed association between AITD and SLE [3]. In a meta-analysis, the prevalence of thyroid autoantibody positivity was higher in patients with SLE compared with healthy controls [4]. However, there was limited evidence on the association between AITD and APS or the presence of aPL. This meta-analysis aimed to provide evidence on the possible association between AITD and the presence of aPL using primary data from all relevant studies.

## 2. Methods

### 2.1. Literature and Search Strategy

A literature search of PubMed, Embase, and Cochrane was conducted for studies from the time when the databases were established to March 2022 with the restriction of English language using the following searching terms: “antiphospholipid antibody” or “antiphospholipid syndrome” in combination with the terms “thyroid diseases” or “Hashimoto's thyroiditis” or “Graves' disease” or “autoimmune thyroid disease.” The studies were filtered for those focused on human subjects. All eligible studies and their eligible references were checked and retrieved. Studies were selected by two independent reviewers, and disagreements were solved after discussion.

### 2.2. Inclusion Criteria

Studies were included in this meta-analysis when they met the following criteria: (1) case-control or cohort design; (2) evaluate the association between AITD and patients with positive aPL; (3) containing sufficient data of cases, healthy controls for calculating the odds ratios (OR), 95% confidence interval (CI), and the *p* value.

### 2.3. Data Extraction and Assessment of Risk of Bias

The following data were extracted from all enrolled studies independently by two of the authors: first author, publication year, numbers of cases of AITD and healthy controls, and numbers of cases and controls with positive aPL.

The Newcastle–Ottawa Scale, recommended by the Cochrane collaboration for risk of bias assessment for observational studies, was applied for all included studies for quality assessments by two independent investigators [5]. Three categories, including the selection of study groups, the comparability between cases and controls, and the certainty of exposure of cases and controls, were judged in all enrolled studies using the Newcastle–Ottawa Scale ranging from zero to nine stars. Furthermore, a funnel plot was conducted for the analysis of potential publication bias.

### 2.4. Statistical Analysis

The association between AITD and positive aPL was investigated by OR with 95% CIs. *p* < 0.05 was considered statistically significant. Heterogeneity was evaluated by chi-squared statistics and *I*^*2*^. Values of 25%, 50%, and 75% were defined as low, moderate, and high heterogeneity, respectively. The random-effects model was used when *I*^*2*^ >50%, indicating heterogeneity across studies. Otherwise, the fixed-effects model was used in the meta-analysis. Funnel plots were performed for analysis of publication bias. All statistical analyses were performed using review manager software (version 5.3).

## 3. Results

### 3.1. Characteristics of the Selected Studies

**Figure 1:**
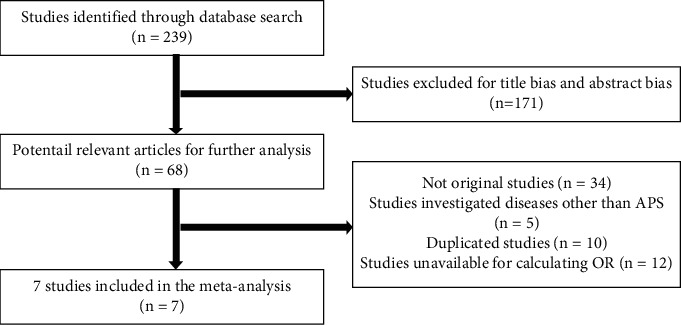
Flowchart of study inclusion and exclusion.

A total of 239 articles were observed during the preliminary search. Among them, 171 articles were excluded after the authors screened the titles and abstracts. The rest of the 68 publications were identified and read in the full article, as shown in Figure 1. Overall, 7 studies that met all inclusion criteria were selected in this meta-analysis, with a total of 475 AITD patients and 764 healthy controls. The characteristics and the rate of positive aPL in AITD patients or healthy controls of each included study in this meta-analysis were summarized in Table 1. The quality assessment for each study in accordance with the Newcastle–Ottawa Scale guidelines is displayed in the S1 table.

### 3.2. Meta-Analysis

**Figure 2:**
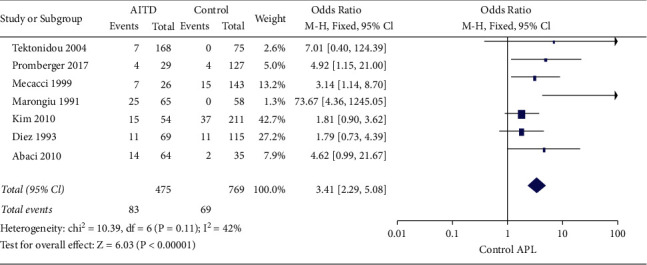
Forest plots for the prevalence of positive aPL in AITD patients compared with healthy controls. AITD: autoimmune thyroid diseases; APL: antiphospholipid antibodies; CI: confidence interval.

In total, 7 studies investigated the prevalence of positive aPL among AITD patients compared with healthy controls [6–12]. The pooled OR with 95% CI was 3.41 (95% CI, 2.29–5.08) (*p* < 0.001), which suggested the risk of positive aPL was approximately 3 folds higher in AITD patients compared with those in healthy controls (Figure 2).

**Figure 3:**
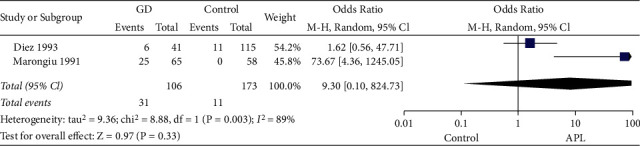
Forest plots for the prevalence of positive aPL in GD patients compared with healthy controls. GD: Graves' disease; APL: antiphospholipid antibodies; CI: confidence interval.

Two studies evaluated the association of GD and positive aPL in comparison to healthy controls [9, 11]. *I*^*2*^ exceeded 50%, thus the random-effects model was applied. The pooled OR with 95% CI was 9.3 (95% CI, 0.10–824.73) (*p*=0.33) (Figure 3), which indicated that there was no significant association between the risk of GD and the presence of aPL.

**Figure 4:**
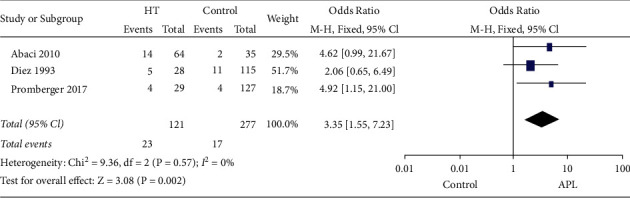
Forest plots for the prevalence of positive APL in HT patients compared with healthy controls. HT: Hashimoto's thyroiditis; APL: antiphospholipid antibodies; CI: confidence interval.

Three studies conducted the research aiming at the relationship between HT and the presence of aPL [7, 11, 12]. The fixed-effects model was used due to the low heterogeneity, and the pooled OR was 3.35 (95% CI, 1.55–7.23) (*p*=0.002) (Figure 4), which demonstrated that the risk of positive aPL in patients with HT increased over 2 folds compared with healthy controls.

### 3.3. Sensitivity Analysis

A sensitivity analysis was performed using sequential omission of the included studies to evaluate the reliability and stability of the conclusions. The pooled ORs were not significantly changed after the analysis.

### 3.4. Publication Bias

Funnel plots were conducted to evaluate the potential publication bias for meta-analysis (Figure S1, Figure S2, and Figure S3). The distribution of the funnel plots suggested no significant publication bias.

## 4. Discussions

To the best of our knowledge, our study is the first systemic review and meta-analysis aiming to explore the association between AITD and the presence of APL. In our study, we discovered the positive association between AITD and positive aPL, as well as HT and the presence of aPL.

AITD, as well as aPS, is common in women of reproductive age that can cause recurrent pregnancy loss and infertility [13]. There are possible mechanisms that could explain several aspects of the association between AITD and recurrent miscarriages. First of all, thyroid dysfunction, especially during the first trimester, results in a worsened reproductive outcome. On the other hand, TAI may induce innate immune responses and affect fetal microchimerism via the immune response against the fetoplacental unit [14]. Also, the acquired immune response was altered, which was presented as T-cell dysfunction in the endometrium and the modulation of implantation [15]. It was demonstrated in a rodent model that antithyroid antibodies could directly bind to the placenta, which might lead to the recurrence of miscarriage [16].

The positivity of aPL antibodies was not explored in patients with AITD, nor was the prevalence of antithyroid antibodies detected in patients with APS. APS could be a primary clinical condition or secondary to other autoimmune diseases including SLE. The positivity rate of antithyroid antibodies in SLE patients has been widely discussed, while the association of both diseases highly varied in the literature [17–19]. Different study designs, the iodine status of the population and regions, and the methods used for antibody measurements contributed to the varying results. Therefore, researchers conducted several meta-analyses to confirm the relationship between SLE and thyroid dysfunction or the positivity of antithyroid antibodies [20, 21]. Luo et al. proved that the prevalence of hypothyroidism in SLE patients was 1.93 folds higher than those in healthy controls, and the risk of subclinical hypothyroidism in SLE patients increased by 4.67 folds compared with healthy controls [20]. Pan et al. revealed that the prevalence of both TPOAb and TgAb in patients with SLE was significantly higher than in healthy controls [21]. A population-based study in Taiwan proved that patients with Graves' disease had a significantly increased risk of SLE (hazard ratio 5.45) [22]. The above studies indicated a positive association between AITD and SLE. However, APS is a rare disease with heterogeneous causes, thus it is difficult to determine the relationship between APS and AITD.

Of all 7 studies included in the meta-analysis, only 1 study evaluated the prevalence of confirmed diagnosed APS in AITD patients and healthy controls in accordance with the classification criteria for definite APS [8]. Therefore, we aimed to explore the risk of the presence of aPL in patients with AITD compared with unaffected individuals using meta-analysis, and a significantly increased risk of positive aPL was discovered in patients with AITD (OR 3.41) and patients with Hashimoto's thyroiditis (OR 3.35). Due to a relatively small number of studies focused on the association between Graves's disease and positive aPL, as well as a high heterogeneity, the relationship between GD and positive aPL was insignificant in our results. In addition, we did systematic research for studies conducted on patients with positive aPL in comparison to healthy controls to identify the risk of AITD in both groups. Two studies were included [23, 24], while there was a lack of data for meta-analysis.

aPL included lupus anticoagulant (LAC), anticardiolipin antibodies (ACL), and anti*β*2-glycoprotein I antibody (anti*β*2-GP I). aPL profile was divided into medium-high titers, high-risk, and low-risk based on different risks for thrombotic and obstetric APS [25]. A high-risk aPL profile indicated the presence of LAC on 2 or more occasions at least 12 weeks apart, or of double or triple aPL positivity (all three subtypes), or the presence of persistently high aPL titers. In this meta-analysis, all 7 included studies measured ACL, while only 1 study detected LAC, and 1 study evaluated anti*β*2-glycoprotein I antibody, thus we failed to conduct further subgroup analysis for different subtypes of aPL or risk for thrombotic and obstetric APS.

Our study is the first meta-analysis to shed light on the association between AITD and the presence of aPL, while there were several limitations to the study. First, due to the lack of consistent diagnostic criteria for APS among different studies, the study population included patients with confirmed diagnosed APS and recurrent miscarriage patients with positive aPL antibodies, which increased the heterogeneity of the meta-analysis. Second, the included studies were conducted by researchers from different countries for the duration of up to three decades, and the diversity of the included studies may lead to possible influence on the study results. Third, several studies with a large study population of positive aPL or AITD were not enrolled in this meta-analysis due to the lack of the comparison of healthy controls or incomplete raw data, thus the study population of our meta-analysis was relatively small. Therefore, the results of this meta-analysis should be interpreted with caution.

In conclusion, the result of this meta-analysis proved that the prevalence of positive aPL was significantly increased in patients with AITD compared with healthy individuals, especially in patients with Hashimoto's thyroiditis. More population-based studies, especially from Asia and other continents, are expected in the future.

## Figures and Tables

**Table 1:** Characteristics of the included studies in this meta-analysis.

Authors	Year	Region	AITD type	AITD cases (*n*/*N*)	Positive rate of aPL (%) in AITD patients	Healthy controls (*n*/*N*)	Positive rate of aPL (%) in healthy controls	The type of aPL
Tektonidou et al. [6]	2004	Greece	AITD	7/168	4.2	0/75	0	ACL

Promberger et al. [7]	2017	Austria	HT	4/29	13.8	4/127	3.1	ACL and/or anti*β*2-GP I antibody

Mecacci et al. [8]	1999	Italy	AITD	7/26	26.9	15/143	10.5	LAC or aCL

Marongiu et al. [9]	1991	Italy	GD	25/65	38.5	0/58	0	ACL

Kim et al. [10]	2010	USA	AITD	15/54	27.8	37/211	17.5	ACL

Diez et al. [11]	1993	Spain	GD (6/41), HT (5/28)	11/69	15.9	11/115	9.6	ACL

Abaci et al. [12]	2010	Turkey	HT	14/64	21.9	2/35	5.7	ACL

*N*: the number of individuals with positive aPL; *N*: the total number of individuals with AITD or healthy controls; AITD: autoimmune thyroid diseases; HT: Hashimoto's thyroiditis; GD: Graves' disease; aPL: antiphospholipid antibodies; aCL: anticardiolipin antibodies; LAC: lupus anticoagulant; anti-*β*2-GP I: anti-*β*2-glycoprotein I antibody.

## Data Availability

Underlying data could be found in supplementary materials.

## Supplementary Materials

Supplementary Materials S1 Fig. Funnel plots of the association between AITD and positive aPL. S2 Fig. Funnel plots of the association between GD and positive aPL. S3 Fig. Funnel plots of the association between HT and positive aPL. S1 table. The quality assessment for each study in accordance with the Newcastle–Ottawa Scale guidelines.
