# Supplementary material for: Patients with Autoimmune Thyroid Diseases Have Higher Prevalence of Positive Antiphospholipid Antibodies: A Systematic Review and Meta-Analysis
Source: Int J Endocrinol. 2022 Aug 27;2022:8271951. doi: 10.1155/2022/8271951 (PMC9440846; doi:10.1155/2022/8271951)
Supplement: Supplementary Materials — S1 Fig. Funnel plots of the association between AITD and positive aPL. S2 Fig. Funnel plots of the association between GD and positive aPL. S3 Fig. Funnel plots of the association between HT and positive aPL. S1 table. The quality assessment for each study in accordance with the Newcastle–Ottawa Scale guidelines. [file 8271951.f1.doc]

Table S1. The quality assessment for the studies using the Newcastle-Ottawa Scale

| Authors | Year | Region | Selection | Comparability | Exposure | Quality Score |
| --- | --- | --- | --- | --- | --- | --- |
| Tektonidou et al. [6] | 2004 | Greece | ★★ | ★ | ★ | ★★★★ |
| Promberger et al. [7] | 2017 | Austria | ★ | ★★ | ★★ | ★★★★★ |
| Mecacci et al. [8] | 1999 | Italy | ★ | ★★ | ★★ | ★★★★★ |
| Marongiu et al. [9] | 1991 | Italy | ★★ | ★ | ★ | ★★★★ |
| Kim et al. [10] | 2010 | USA | ★ | ★★★ | ★★ | ★★★★★★ |
| Diez et al. [11] | 1993 | Spain | ★ | ★★★ | ★★ | ★★★★★★ |
| Abaci et al. [12] | 2010 | Turkey | ★★ | ★★ | ★★ | ★★★★★★ |

All included studies were judged based on three categories including the selection of study groups, the comparability of cases and controls, and the exposure for cases and controls, using the star system.

Figure S1. Funnel plots of the association between AITD and positive APL. AITD, autoimmune thyroid diseases; APL, antiphospholipid antibodies; OR, odds ratio.

Figure S2. Funnel plots of the association between GD and positive APL. GD, Graves’ disease; APL, antiphospholipid antibodies; OR, odds ratio.

Figure S3. Funnel plots of the association between HT and positive APL. HT, Hashimoto’s thyroiditis; APL, antiphospholipid antibodies; OR, odds ratio.
